# Intermittent suckling with or without co-mingling of non-littermate piglets before weaning improves piglet performance in the immediate post-weaning period when compared with conventional weaning

**DOI:** 10.1186/s40104-017-0144-x

**Published:** 2017-01-28

**Authors:** Diana L. Turpin, Pieter Langendijk, Kate Plush, John R. Pluske

**Affiliations:** 1 School of Veterinary and Life Sciences, Murdoch University, Murdoch, WA 6150 Australia; 2 Trouw Nutrition, Veerstraat 38, Boxmeer, 5831 JN The Netherlands; 3 South Australian Research and Development Institute, Roseworthy Campus, JS Davis Building, Roseworthy, SA 5371 Australia; 4 Present address: SunPork Farms, 563 Coleman Road, Pinkerton Plains, SA 5400 Australia

**Keywords:** Behaviour, Co-mingling, Intermittent suckling, Piglet, Weaning

## Abstract

**Background:**

In this experiment, intermittent suckling (IS) with or without the co-mingling (CoM) of piglets was studied as a method to stimulate solid feed intake and reduce post-weaning stress.

**Methods:**

Three weaning regimes using 30 multiparous sows were compared: (1) conventional weaning (CW) (*n* = 10 litters), where piglets had continuous access to the sow until weaning (d 0, farrowing = d −25 relative to weaning); (2) intermittent suckling (IS) (*n* = 10 litters), where piglets were separated from the sow for 8 h/d starting at d −7 (relative to weaning); and (3) intermittent suckling with co-mingling (ISCo) (*n* = 10 litters) where IS started at d −7 and two litters were housed together during separation and then returned to their original sow. Ad libitum creep feed was available from d −17. At weaning pigs were housed in pens of 11 pigs, 27 pens in total. The ISCo treatment was divided in half to examine effects of different mixing strategies after weaning. Half of the ISCo litters were kept in familiar groups (ISCoF, familiar, *n* = 4) and the other half were mixed within treatment resulting in groups of unfamiliar pigs (ISCoNF, not familiar, *n* = 5), the same as IS (*n* = 9) and CW (*n* = 9) treatments.

**Results:**

The ISCo piglets ate more creep feed in the week before weaning (*P* < 0.01), but also showed more aggressive and manipulative behaviour on first day of CoM compared with CW piglets (*P* < 0.05). IS with or without CoM increased exploratory and play behaviour on the first day of treatment intervention (*P* < 0.001) and increased sleeping behaviour on the last day of treatment intervention compared with CW (*P* < 0.001). Mixing strategy at weaning had an effect on performance data with the highest growth and feed intake seen in ISCoF pigs 2 to 8 d after weaning (*P* <0.001). IS and ISCoNF pigs also grew faster and ate more than CW pigs 2 to 8 d after weaning (P < 0.001). Post-weaning injury scores suggested reduced aggression in ISCo as evidenced by reduced redness (skin irritation) (*P* < 0.05), and a tendency for ISCo to have less scratches than CW (*P* < 0.1). The IS pigs slept the most and displayed less manipulative behaviours on the day of weaning and plasma haptoglobin levels remained low in IS pigs after weaning (*P* ≤ 0.01).

**Conclusions:**

Both intermittent suckling techniques improved production indices in the immediate post-weaning period. However, the addition of co-mingling before weaning in combination with grouping familiar pigs together after weaning improved performance in an additive manner.

## Background

Under current commercial pork production conditions, weaning is an abrupt transition to independency that takes place at 21 to 28 d of age. Despite efforts to familiarise piglets with creep feed during lactation, there is often large within and between litter variation in intake [1]. A lack of familiarity with feed, in combination with other stressors such as a change in housing, maternal separation and mixing with unfamiliar piglets, generally causes a period of reduced nutrient intake immediately after weaning [2, 3]. To avoid a consequential reduction in growth and gastrointestinal tract (GIT) inflammation and dysfunction that generally results from underfeeding in combination with other weaning-associated stressors [4, 5], there has been a large focus on finding methods that stimulate feeding behaviour in this period.

Intermittent suckling (IS), a gradual weaning regime that also mimics the increasing time a sow would spend away from her piglets under natural conditions, has shown an improvement in post-weaning feed intake and growth in litters compared with conventional weaning [6, 7]. This improvement in post-weaning performance is likely mediated through increase familiarisation with creep feed as piglets are forced to explore sources of nutrition other than milk. Alternatively, habituation with maternal separation may also prevent or attenuate the weaning-associated stress response [8], reducing the development of altered behaviour patterns such as aggression, manipulation and a lack of play behaviour in recently weaned piglets [9]. With a worldwide shift towards housing systems that reduce sow confinement, IS has also received renewed interest from a reproductive point of view as a potential way to mate sows during lactation rather than after weaning [10].

Piglets will also generally have a higher solid feed intake after weaning if they are kept in alternative housing that resembles natural conditions, such as multi-suckling and get-away systems with a communal piglet area [11–13]. In a study that used a get-away system with a communal piglets area, sows spent 14 h/d away from the piglets by the end of lactation (d 27) and piglets spent 40% of the observation time in the pens of other litters [11]. By allowing non-littermates to co-mingle (CoM) in these systems, piglets are more likely to have positive play experiences [14] and improved social skills, which results in better acceptance of unfamiliar pigs and reduced aggression after weaning [15–17].

In this study, we developed a novel IS system to include the opportunity for pre-weaning socialisation (CoM), where non-litter mates could interact with each other before weaning to potentially improve social development and reduce post-weaning aggression. We hypothesised that piglets subjected to IS (8 h separation per day for 7 d) would show better growth, higher feed intake, reduced negative behaviour patterns and reduced blood markers of inflammation, stress and lipid mobilisation supporting an increase in more feed directed behaviours before and after weaning. Intermittent suckling was applied in two regimes, with and without CoM (the mixing of piglets from two litters during separation) and compared with conventional weaning. It was expected that combining IS with CoM would improve post-weaning performance in an additive manner and further reduce aggressive behaviour in the immediate post-weaning period.

## Methods

This study was conducted at a commercial piggery in Western Australia. At the conclusion of the study, the pigs continued on a grower/finisher facility at another location.

### Animals, housing and diet

A total of 30 primiparous and multiparous sows (Large White x Landrace) and their offspring were selected based on their farrowing date from the farm and used in a single replicate. One week before farrowing, pregnant sows were housed individually across three adjacent rooms consisting of 5, 16 and 9 sows, respectively, with parities ranging from one to seven.

Individual housing consisted of farrowing crates (0.6 × 2.4 m) within farrowing pens (1.8 × 2.4 m) with slatted flooring. The pens consisted of a covered, heated creep area with artificial lighting provided between 0700 and 1700 h, a single feeder, and two nipple waterers. Water was provided on an ad libitum basis. An average litter size of 10.7 ± 0.48 (mean ± SD) was achieved by cross-fostering within the first 3 d of farrowing within each pre-allocated treatment group. Within 1 week of farrowing, piglets were made individually identifiable with numbered ear tags, their tails were docked, males were castrated and all piglets received a 1 ml intramuscular (IM) iron injection (PigDex100, Aventis Animal Nutrition, Carole Park, QLD, 4300) and a 2 mL IM injection of Respisure One® (*Mycoplasma hypopneumoniae* vaccine; Pfizer, West Ryde, NSW, 2114). The beginning of the experiment, d −25 (25 d before weaning), was designated as the day on which most litters were born. Litters were born from 1 d before to 1 d after d −25.

Sows were fed a standard lactation diet ad libitum from entry into the farrowing house (approximately 1 wk before farrowing) until weaning (14.5 MJ/kg digestible energy (DE); crude protein (CP), 19.1%). Piglets were offered creep feed (15 MJ/kg DE; CP, 23%) *ad libitum* from a single rotary hopper feeder (27 cm diameter) from 17 d before weaning (d −17). Immediately preceding weaning, piglets received a second 2 mL IM dose of Respisure One® (*Mycoplasma hypopneumoniae* vaccine; Pfizer, West Ryde, NSW, 2114) as well as a 2 mL IM injection of Relsure® PCV (Porcine circovirus Type 1 vaccine, Zoetis, Florham Park, NJ, 07932). At weaning, piglets were moved to a separate building and placed into weaner pens with slatted flooring (1.13 m × 2.5 m) in a temperature controlled building. Feed was provided on an ad libitum basis (14.6 MJ/kg DE; CP, 20.8%) via a 5-hole weaner hopper. Piglets remained in the weaner pens until the end of the experiment. Pigs had unlimited access to drinking water provided by a single water nipple in each pen.

### Experiment design

Once farrowing was completed, selected sows and their litters were randomly allocated to one of three treatments: conventional weaning (CW) (*n* = 10 litters), IS (intermittent suckling) (*n* = 10 litters) or ISCo (intermittent suckling with CoM) (*n* = 10 litters). Litters receiving different treatments were spread evenly across the three adjacent rooms and parity remained similar across the three treatments (3.6 ± 2.1 for CW, 3.5 ± 2.0 for IS and 3.6 ± 2.0 for ISCo; mean ± SD). The piglets in the CW treatment remained with the sow continuously through lactation until weaning. The piglets in the IS and ISCo treatments were separated from their sow and housed in an empty farrowing crate in another room for 8 h/d (0700 to 1500 h) for 7 d before weaning. Separation was achieved by transporting piglets by litter in a trolley from one room to another. The empty farrowing pens in which separated piglets were housed were identical to those that housed the sows, but no sows were housed in the separation room. For the ISCo treatment, two litters were housed in one farrowing pen during the separation time to allow for pre-weaning socialisation. The space allowance was 0.42 m^2^ and 0.21 m^2^ for IS and ISCo piglets respectively. In the ISCo treatment, the same two litters were socialised each day and the original litter was returned to the sow at the end of separation. When piglets were separated from the sow, the rotary feeder with creep feed was moved with the litter into the separation pen. The separation pens housing the ISCo piglets therefore had two rotatory feeders per pen during separation.

Weaning age across treatments was equal and averaged 25.3 ± 0.7 (mean ± SD). Piglets were mixed and housed in pens of 10 to 13 with an average of 11.1 ± 0.82 (mean ± SD) per pen. Mixing for CW and IS was done within treatment by randomly allocating 2–3 piglets per litter into each pen. The grouping of ISCo piglets after weaning was achieved in one of two ways: (i) four litters were only grouped with piglets they had previously had contact with before weaning (called ISCoF; familiar), and (ii) six litters were mixed with unfamiliar piglets (similar to CW and IS) (called ISCoNF; not familiar). This arrangement was necessary to examine the effect of familiarity on post-weaning behaviours since reduced aggression in pigs socialised before weaning has been partly attributed to a higher number of familiar pigs in the group rather than previous social experience [18].

### Measurements

#### Body weights, feed intake and injury scores

All piglets were individually weighed on d −17, −7 and −4 before weaning, at weaning, and on d 2 and 8 after weaning. Creep and weaner feed residuals were measured simultaneously with body weights. Minimal wastage was observed due to twice daily checking of the feeders by staff to ensure the pan was not too full. Therefore, disappeared creep feed was considered eaten.

During the weighing procedure on the day of weaning (d 0) and 2 d after weaning, an injury score was recorded from all piglets as an indicator of aggression. The injury scoring system was adapted from Widowski et al. [19] and consisted of a four-point scale for scratches and redness around the head, ears and flank (Table 1).

**Table 1:** Injury scoring system using scratches and redness adapted from Widowski et al. [19]

Score	0	1 (Mild)	2 (Moderate)	3 (Severe)
Scratches	No scratches were evident on the head, ears or flank	1 to 3 small (≤2 cm) scratches or areas of abraded skin on head, ears or flank	1 to 3 large (>2 cm) scratches or areas of abraded skin on head, ears or flank	More than 3 scratches or larger areas of superficial skin loss on head, ears or flank
Redness	No redness or swelling on the head, ears or flank	Redness or swelling barely detectable on head, ears or flank	Redness or swelling were obvious on head, ears or flank	Irritation easily observed as darker reddening and/or moderate to severe swelling on head, ears or flank

### Behavioural measurements

At the beginning of the experiment, five focal piglets were randomly selected from each litter (a total of 150 piglets, 50 piglets per treatment group) and made individually identifiable with marker spray. Instantaneous scan sampling by one observer was then used to record the main activity of the individual selected piglets on d −10, −7, −1, 0, 1 and 7. All focus piglets were observed every 30 min for two, 2-h periods (morning 0900 and afternoon 1400), excluding d 0 and d 1 during which only one, 2- h period was used to record behaviour. The different types of behaviour recorded during scan sampling were adapted from behaviour categories previously used by Pluske and Williams [20] and Bolhuis et al. [21] and are presented in Table 2.

**Table 2:** Ethogram used during instantaneous scan sampling observations adapted from Pluske and Williams [20] and Bolhuis et al. [21]

Behaviour	Description
Sleeping behaviour	Lying on the side or belly with eyes closed, not performing any other described behaviour
Lying/sitting behaviour	Lying on the side or belly with eyes open or passive sitting, not performing any other described behaviour
Standing behaviour	Standing without performing any other described behaviour
Aggressive behaviour	Head knocking, biting or fighting with another pen or littermate
Exploring/play behaviour	Standing up and investigating the surroundings such as nosing the floor, scrapping the floor with one of the forelegs, nosing or nibbling on fixtures. Running across the pen and pivoting with or without the gentle nudging of a pen or littermate
Manipulative behaviour	Belly nosing Mounting Oral manipulation of other pen or littermates Laying down and biting the metal frame work of the weaner pens (after weaning only)
Ingestive-related behaviour	Eating (chewing feed) Drinking from water nipple Eliminating (defecating or urinating)
Sow directed behaviour	Suckling or massaging the sow Manipulation of the sow during the pre-weaning period

### Plasma analysis

Blood samples were taken from two randomly selected piglets per litter or pen on d −6 and −4 before weaning and d 2 and 4 after weaning (piglets were not bled more than two times within a 7 d period). Piglets were held in dorsal recumbancy and blood samples were collected via jugular venipuncture with the procedure lasting no more than 90 s. Nine millilitres of blood was collected into lithium heparin coated tubes. Blood samples were taken at the same time of day (noon) to minimise the effects of diurnal variation. Plasma was separated by centrifugation (20 min at 2,800 × g, 4 °C) and then stored as 0.5 mL aliquots at −20 °C until analysis.

Plasma cortisol levels were determined using a commercial ELISA kit (Enzo Life Sciences, Cortisol ELISA kit, AD-901-071, Farmingdale, NY) in accordance with the manufacturers’ instructions with the exception of optical density, which was read at 415 nm instead of the recommended 405 nm. Intra-assay CV was 10.5% (low standard), 6.6% (medium standard) and 7.3% (high standard). Plasma was analysed at Animal Health Laboratories (Department of Agriculture and Food Western Australia) for the determination of (i) haptoglobin (Hp), using an enzymatic colorimetric in-house assay based on modified methods of Eckersall et al. [22], and (ii) glycerol, using a Randox Reagent Kit (GY105, Crumlin, United Kingdom) and Olympus AU400 analyser (Tokyo, Japan).

### Statistical analysis

Statistical analyses were performed using SPSS (v.21; IBM). Residuals were tested for normality and data were transformed or non-parametric tests were used if needed. Analysis of post-weaning data included four treatment groups after weaning (CW, IS, ISCoF and ISCoNF; as described above). Differences between ISCoF and ISCoNF were present for post-weaning ADG (average daily gain), ADFI (average daily feed intake) and FCR (feed conversion ratio) (*P* < 0.05). Therefore, post-weaning performance data are presented with four treatment groups (CW, IS, ISCoF and ISCoNF). However, no differences between ISCoF and ISCoNF were present for behaviour, injury score or blood parameter data (*P* > 0.05; with the exception of sleeping behaviour on d 7 after weaning during which ISCoNF slept more than ISCoF, *P* < 0.01). Therefore, analysis was repeated with three treatment groups for post-weaning data for these parameters (CW, IS and ISCo).

Data for pre-weaning piglet mortality, body weight (BW), ADG, ADFI and FCR were analysed on a per litter or pen basis using a general linear model with treatment as the fixed factor. Since two feeders were included in each of the ISCo separation pens, ADFI data for the ISCo treatment group before weaning were analysed on a per socialised group basis (i.e. *n* = 5). To normalise the distribution of pre-weaning ADFI data, feed disappearance at −7 to −4 d before weaning and −4 to 0 d before weaning were pooled together and presented as feed disappearance during the last week of lactation. Unexpectedly, feed disappearance from d −17 to d −7 for the IS treatment groups tended to be higher than that of the controls (9 ± 1.7, 8 ± 2.3 and 3 ± 1.6 g/piglet/day for IS, ISCo and CW respectively, *P* < 0.1), therefore, ADFI from d −17 to d −7 was included as a covariate in the analysis of the pre-weaning ADFI data. Feed conversation ratio was calculated by dividing ADFI (d 2 to 8) by ADG (d 2 to 8).

Measurements for injury scores, plasma cortisol, Hp and glycerol were averaged per crate or pen and then analysed using a general linear model with treatment as a fixed factor. Redness scores before and after weaning, as well as plasma Hp concentration were not normally distributed. Data were transformed using a square root transformation to force normality. The mean values and confidence intervals were then back-transformed and expressed as least square means.

Behaviour data obtained from instantaneous scan sampling are expressed as a percentage of total observations for each behaviour on a specific day. The distribution of all behaviours observed was not normal and transformation of the data did not correct this. The percentage of scan samples for a specific behaviour was therefore compared between treatments on different days using a Kruskal-Wallis test with post-hoc analysis to determine which groups were different from one another. A non-parametric Friedman test was used to compare differences in proportion of total observations spent on a specific behaviour within treatment. If this test detected an overall treatment effect, data were subsequently tested pairwise using a Wilcoxon test.

All post-hoc analyses included a Bonferroni correction for pairwise comparisons and correlations were performed using a Pearson correlation test. Statistical significance was accepted at *P* ≤ 0.05 and a trend was considered at *P* > 0.05 and *P* ≤ 0.1. Data are presented as raw means ± SEM, except when *n* is different between treatments, in which case data are presented as raw means ± SE unless otherwise stated.

## Results

### Piglet mortality

Pre-weaning piglet mortality was similar between all treatments (*P* > 0.05). Mortality mostly occurred before treatment intervention (CW: 0.4 ± 0.70, IS: 0.3 ± 0.67, ISCo: 0.1 ± 0.32 piglets per litter, *P* > 0.05). Litter sizes on the first day of treatment intervention did not differ (CW: 10.4 ± 0.70, IS: 10.3 ± 0.95, ISCo: 10.5 ± 0.53, *P* > 0.05) and mortality after the start of IS with or without CoM was negligible, with only 1 piglet in the IS treatment group dying due to crushing on d −4 of the experiment. Therefore, litter sizes at weaning were 10.4 ± 0.70 for CW, 10.2 ± 1.23 for IS and 10.5 ± 0.53 for ISCo (*P* > 0.05). None of the treatment groups experienced post-weaning mortalities, however, 9 piglets were not weaned due to having a BW less than 4.6 kg (5 piglets from CW, 1 piglet from IS and 3 piglets from ISCo) and were returned to the herd with a nurse sow as per regular farm protocol.

### Production data

Piglet BW and ADG were similar between all treatments between d −17 to −7 (Table 3, *P* > 0.05). During the last week of lactation (d −7 to 0), exposure to IS or IS with CoM did not affect BW or growth compared with CW litters (Table 3, *P* > 0.05). Therefore, BW did not differ between the three treatment regimes at weaning (Table 3, *P* > 0.05). However, ISCo litters ate more creep feed than IS and CW litters between d −7 and 0 (Table 3, *P* < 0.01).

**Table 3:** Mean values for piglet BW, ADG and ADFI before weaning for three different weaning treatments

	Treatment^2^			
Day^1^	CW	IS	ISCo	SEM
BW, kg				
D −17	2.8	2.9	3.0	0.14
D −7	5.4	5.7	5.6	0.22
D −4	6.3	6.4	6.4	0.22
D 0	7.3	7.4	7.5	0.23
ADG, g				
D −17 to −7	265	278	272	8.76
D −7 to −4	283	229	270	19.44
D −4 to 0	267	253	251	12.84
ADFI^3^,g				
D −7 to 0	7^a^ ± 2.1	15^a^ ± 2.1	22^b^ ± 3.0	

^1^Day = day in relation to weaning with weaning = d 0, d −25 is the day on which most the litters were born

^2^CW = conventional weaning (*n* = 10), IS = intermittent suckling (*n* = 10), ISCo = intermittent suckling with co-mingling (*n* = 10)

^3^D −17 to −7 ADFI included as covariate in analysis

^ab^Values within a row not having the same superscript are significantly different

After weaning, all treatment groups suffered a reduction in weight gain from d 0 to 2. Between d 2 and 8, ISCoF pens grew the fastest, CW pens grew the slowest and IS and ISCoNF pens were intermediate (Table 4, *P* < 0.001). At the end of the experiment (d 8), BW was similar across all four treatment groups (Table 4, *P* > 0.05).

**Table 4:** Mean values for pig BW, ADG, ADFI and FCR after weaning for three different weaning treatments

Day^1^	Treatment^2^			
	CW	IS	ISCoF	ISCoNF
BW, kg				
D 2	7.5 ± 0.23	7.3 ± 0.23	7.3 ± 0.34	7.6 ± 0.31
D 8	8.4 ± 0.24	8.7 ± 0.24	9.0 ± 0.37	9.0 ± 0.33
ADG, g				
D 0 to 2	−25 ± 13.9	−56 ± 13.7	−78 ± 21.6	−48 ± 17.7
D 2 to 8	165 ± 8.6^a^	234 ± 8.5^b^	294 ± 13.4^c^	242 ± 11.1^b^
ADFI, g				
D 0 to 2	80 ± 5.3	73 ± 5.3	67 ± 7.9	72 ± 7.1
D 2 to 8	192 ± 4.5^a^	256 ± 4.5^b^	291 ± 6.7^cy^	269 ± 6.0^bcx^
FCR, g/g				
D 2 to 8	1.2 ± 0.03^a^	1.1 ± 0.03^ab^	1.0 ± 0.05^b^	1.1 ± 0.04^ab^

^1^Day = day in relation to weaning with weaning = d 0, d −25 is the day on which most the litters were born

^2^CW = conventional weaning (*n* = 9), IS = intermittent suckling (*n* = 9), ISCoF = intermittent suckling with co-mingling, familiar pigs (*n* = 4), ISCoNF = intermittent suckling with co-mingled, not familiar pigs (*n* = 5)

^ab^Values within a row not having the same superscript are significantly different

^xy^Values within a row not having the same superscript are a trend

Weaning markedly increased feed intake in all treatment groups relative to intake in lactation, however ADFI did not differ between treatments during the first 2 d after weaning. Between d 2 to 8 after weaning, all IS treatment pens were eating more than CW pens (Table 4, *P* < 0.001), however, ISCoF were eating more than IS pens (*P* < 0.001) and there was a tendency for ISCoF to eat more than ISCoNF pens (P < 0.1). Feed conversion ratio was lower for ISCoF than CW between 2 and 8 days after weaning, while IS and ISCoNF were intermediate (Table 4, *P* < 0.05).

### Behavioural measurements

#### Pre-weaning behaviours

Before the start of IS intervention (d −10), there was no difference (*P* > 0.05) between treatments in the proportion of total observations spent per behaviour category (Table 5). On d −7, treatment intervention caused an increase in lying and sitting behaviour for both IS and ISCo piglets compared with d −10 behaviour observations for each treatment (Table 5, *P* < 0.001). Furthermore, exploratory and play behaviour also increased (*P* < 0.001) in IS piglets between d −10 to −7 whereas aggressive behaviour increased in ISCo piglets (*P* < 0.001). Compared with CW piglets, ISCo piglets showed a greater proportion of standing, manipulative and aggressive behaviours on d −7 while IS piglets were intermediate (Table 5, *P* < 0.05 for all behaviours). Only piglets subjected to ISCo slept more, had higher levels of other inactive behaviour (lying and sitting) and showed a greater proportion of total observations on ingestive-related behaviours compared with IS and CW piglets on d −7 (Table 5, *P* ≤ 0.001 for all behaviours).

**Table 5:** Pre-weaning behaviour (proportion of total observations, %) of selected CW, IS and ISCo piglets^1^

Behaviour^2^, % of total observations	D^3^ -10				D −7				D −1			
	CW	IS	ISCo	SEM	CW	IS	ISCo	SEM	CW	IS	ISCo	SEM
Sleeping	50.5	44.0^x^	39.0^x^	3.9	54.8^a^	55.0^a,y^	24.5^b,y^	2.7	47.8^a^	91.2^b,z^	95.0^b,z^	2.3
Inactive	2.0^x^	5.5^x^	3.0^x^	1.3	4.5^a,xy^	13.5^b,y^	32.5^c,y^	1.6	7.0^a,y^	3.0^b,x^	0.5^b,z^	1.6
Standing	6.0	7.5^x^	5.5^x^	1.8	4.3^a^	6.3^ab,x^	7.8^b,x^	1.1	2.0^a^	0.3^b,y^	0.0^b,y^	0.4
Aggressive behaviour	3.0	2.0	0.5^x^	1.1	0.5^a^	1.0^ab^	3.0^b,y^	0.6	0.0	0.0	0.3^x^	0.1
Exploratory and play behaviour	6.0	10.0^x^	12.0^x^	2.1	7.8^a^	17.0^b,y^	16.8^b,x^	1.9	7.3^a^	1.5^b,z^	0.5^b,y^	0.9
Manipulative behaviour	4.5	6.0	5.5^x^	1.7	3.0^a^	4.5^ab^	7.5^b,x^	1.2	3.0^a^	0.8^b^	0.5^b,y^	0.6
Ingestive-related behaviour	4.0	5.0	5.5	1.5	2.3^a^	1.0^a^	5.8^b^	0.9	3.8	3.2	3.0	1.0
Sow-directed behaviour	24.0	20.0	29	3.1	22.3	-	-	1.2	28.5	-	-	1.3

^1^CW = conventional weaning (*n* = 50), IS = intermittent suckling (*n* = 50), ISCo = intermittent suckling with co-mingling (*n* = 50)

^2^Results obtained by scan sampling observations. Data were tested nonparametrically

^3^Day = day in relation to weaning with weaning = d 0, d −25 is the day on which most the litters were born

^a-c^For each day, means with different superscripts are different (*P* < 0.05), indicating differences between treatments per day

^x-z^For each day treatment, means with different superscripts (*P* <0.05), indicating differences between days within each treatment

Over the course of treatment intervention (d −7 to −1), sleeping increased in both IS and ISCo piglets (*P* < 0.001) with piglets from both IS treatments spending over 90% of total observations during separation sleeping in their crates, which was higher than the level of sleeping for CW piglets (*P* < 0.001). Consequently, the expression of all other behaviour categories reduced over time (d −7 to −1) for ISCo (*P* < 0.001) and lying/sitting, standing and exploratory and play behaviour reduced over time (d −7 to −1) for IS piglets (*P* < 0001). As a result, observations for lying/sitting (*P* < 0.001), standing (*P* < 0.001), exploration/play (*P* < 0.001) and manipulation (*P* < 0.01) were lower in IS and ISCo piglets on day −1 compared with CW piglets (Table 5). There was no difference in the expression of ingestive-related behaviour or aggressive behaviour between treatments on day −1 (Table 5, *P* < 0.05).

#### Post-weaning behaviours

On the day of weaning (approximately 3 h after the event), a considerable proportion of observations was spent on sleeping behaviour in IS pigs with IS pigs sleeping more than CW and ISCo pigs (*P* < 0.001) and CW and ISCo pigs lying or sitting more than IS pigs (Table 6, *P* < 0.001). More manipulative and exploratory/play behaviour was also observed for both CW and ISCo pigs compared with IS pigs (*P* < 0.001) on the day of weaning and CW pigs displayed the highest level of standing behaviour compared with both IS treatments (Table 6, *P* < 0.001).

**Table 6:** Post-weaning behaviour (proportion of total observations, %) of CW, IS and ISCo pigs^1^

Behaviour^2^, % of total observations	D^3^ 0				D 1				D 7			
	CW	IS	ISCo	SEM	CW	IS	ISCo	SEM	CW	IS	ISCo	SEM
Sleeping	20.4^a,x^	91.3^b,x^	22.4^a,x^	2.8	53.1^a,y^	58.2^a,y^	78.0^b,y^	4.1	39.8^a,z^	58.9^b,y^	54.6^b,z^	3.1
Inactive	32.1^a,x^	0.5^b,x^	18.4^c,x^	2.7	19.9^a,y^	15.8^a,y^	8.1^b,y^	2.3	15.1^a,y^	6.6^b,z^	11.2^ab,y^	1.4
Standing	13.3^a,x^	2.6^b^	4.1^b^	1.9	4.6^y^	6.6	5.1	1.9	8.7^x^	4.8	4.8	1.3
Aggressive behaviour	2.0	0.0	1.5	0.9	0.0	0.0	0.0	0.0	0.0	0.8	0.0	0.0
Exploratory and play behaviour	15.8^a,x^	0.0^b,x^	31.6^ax^	2.5	5.6^y^	5.1^y^	2.6^y^	1.5	8.2^y^	6.6^y^	3.1^y^	1.5
Manipulative behaviour	10.7^a,xy^	1.0^b,x^	12.8^a^	2.1	7.7^ax^	4.6^ab,x^	3.1^b^	1.5	13.0^y^	10.7^y^	15.3	1.8
Ingestive-related behaviour	2.0^a,x^	1.5^a,x^	8.7^b,x^	1.3	6.6^y^	9.2^y^	3.1^y^	2.1	13.0^z^	9.9^y^	11.0^x^	1.5

^1^CW = conventional weaning (*n* = 50), IS = intermittent suckling (*n* = 50), ISCo = intermittent suckling with co-mingling (*n* = 50)

^2^Results obtained by scan sampling observations. Data were tested nonparametrically

^3^Day = day in relation to weaning with weaning = d 0, d −25 is the day on which most the litters were born

^a-c^For each day, means with different superscripts are different (*P* < 0.05), indicating differences between treatments per day

^x-z^For each day treatment, means with different superscripts (*P* <0.05), indicating differences between days within each treatment

Over the initial 24 h after weaning (d 0 to d 1), sleeping and lying/sitting behaviour varied differently over time for each of the different treatments (see Table 6) resulting in higher levels of sleeping for ISCo than CW and IS pigs and higher levels of lying or sitting behaviour for CW and ISCo pigs than IS pigs 24 h after weaning (Table 6, *P* < 0.001). While the proportion of total observations spent on manipulative behaviour did not change for CW and IS pigs, there was a reduction in observations for ISCo pigs over the initial 24 h after weaning (Table 6, *P* < 0.001). Therefore, on d 1 after weaning, ISCo pigs expressed less manipulative behaviour than CW pigs, and IS pigs were intermediate (*P* < 0.05). Alternatively, exploratory and play behaviour increased over the initial 24 h after weaning for IS pigs while the proportion of total observations for exploratory and play behaviour decreased over the same timeframe for ISCo and CW pigs (*P* < 0.001 for IS and ISCo and *P* =0.001 for CW).

Apart from the day of weaning during which ISCo pigs had the highest level of eating behaviour (Table 6, *P* < 0.001), no difference in eating patterns were observed between treatments after weaning (*P* > 0.05).

One week after weaning, the proportion of total observations spent on sleeping in both IS treatments was more than the CW pigs (*P* < 0.001), but lying and sitting was less in both IS treatments compared with CW pigs (*P* = 0.001). In contrast, the proportion of total observations spent on the other behaviour categories was similar for all treatments 7 d after weaning (Table 6, *P* > 0.05), with the exception of aggressive behaviour where IS pigs fought more than CW and ISCo pigs (*P* > 0.05). However, this type of behaviour was still rarely observed.

### Injury scores

On the day of weaning, there was no difference in scratch scores between treatments (Table 7, *P* > 0.05), however CW piglets had higher redness scores than piglets in either IS treatment (Table 7, *P* < 0.05). Two days after weaning, there was a tendency for pigs in CW pens to have more scratches than pigs in ISCo pens (Table 7, *P* < 0.1). At the same point in time, higher redness scores were seen in CW and IS pigs compared with ISCo pigs (Table 7, *P* < 0.001).

**Table 7:** Effects of three different weaning treatments on injury score on the day of weaning (just prior to the process of weaning) and at 2 d after weaning

	Treatment^1^				
	CW	IS	ISCo	SEM	*P*-value
Scratch injury score					
Weaning^2^	0.48	0.30	0.51	0.09	0.237
D 2 after weaning	1.03^x^	0.97^xy^	0.55^y^	0.15	0.074
Redness injury score^3^					
Weaning^2^	0.21^a^(0.11-0.35)	0.05^b^(0.01-0.13)	0.04^b^(0.01-0.12)		0.016
D 2 after weaning	0.48^a^(0.32-0.67)	0.35^a^(0.21-0.52)	0.02^b^(0.00-0.08)		<0.001

^1^CW = conventional weaning (*n* = 10 pre-weaning, *n* = 9 post-weaning), IS = intermittent suckling (*n* = 10 pre-weaning, *n* = 9 post-weaning), ISCo = intermittent suckling with co-mingling (*n* = 10 pre-weaning, *n* = 9 post-weaning)

^2^Injury scores were assessed just prior to weaning

^3^Data were square-root transformed and then back transformed and expressed as least square means with 95% confidence intervals

^ab^Values within a row not having the same superscript are significantly different

^xy^Values within a row not having the same superscript are a trend

Before weaning, scratch scores did not correlate with redness scores (*r* = 0.11, P > 0.05). However, scratch and redness scores were positively correlated after weaning (*r* = 0.67, *P* < 0.001). There was a weak positive correlation across treatments between aggressive behaviour the day before weaning (d −1) and scratch scores measured on the day of weaning (just prior to the event of weaning) (*r* = 0.22, *P* < 0.01).

### Blood measures

Pre-weaning treatment did not affect plasma cortisol concentration before weaning and concentrations were still similar between treatments 2 d after weaning (Table 8, *P* > 0.05). Four days after weaning, cortisol levels for IS pigs were higher than that of ISCo pigs (Table 8, *P* = 0.001) and tended to be higher than that of CW pigs (Table 8, *P* < 0.1).

**Table 8:** Plasma cortisol (ng/ml) concentrations during three different weaning treatments before and after weaning

Day^1,2^	Treatment^3^			*P*-value
	CW	IS	ISCo	
D −6	19 (11.0-29.6)	19 (10.6-28.8)	23 (14.1-34.6)	0.724
D −4	20 (12.1-29.6)	27 (18.0-38.5)	22 (13.4-31.7)	0.467
D 2	28 (14.9-45.5)	20 (9.2-35.0)	24 (12.2-40.7)	0.672
D 4	25^abx^(17.9-32.6)	32^axy^(24.4-41.1)	14^by^(8.9-19.8)	0.001

^1^Day in relation to weaning with 0 representing weaning (e.g. 2 is 2 d after weaning)

^2^Data has been transformed via square root and then back transformed and expressed as least square means with 95% confidence intervals

^3^CW = conventional weaning (*n* = 10 pre-weaning, *n* = 9 post-weaning), IS = intermittent suckling (*n* = 10 pre-weaning, *n* = 9 post-weaning), ISCo = intermittent suckling with co-mingling (*n* = 10 pre-weaning, *n* = 9 post-weaning)

^ab^Values within a row not having the same superscript are significantly different

^xy^Values within a row with different superscripts are trends

**Fig. 1:**
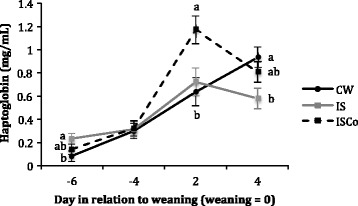
Plasma haptoglobin (mg/mL) concentrations in the three different weaning treatments before and after weaning. Values are presented as actual means ± SE. Analysis involved transformation of the data using a square root calculation; CW = conventional weaning (*n* = 10 pre-weaning, *n* = 9 post-weaning), IS = intermittent suckling (*n* = 10 pre-weaning, *n* = 9 post-weaning), ISCo = intermittent suckling with co-mingling (*n* = 10 pre-weaning, *n* = 9 post-weaning). ^a,b^ On each experimental day, values not having the same superscript are significantly different.

Plasma Hp concentrations were higher in IS piglets compared to CW piglets on d −6 before weaning (the second day of IS intervention), and ISCo Hp levels were intermediate (Fig. 1, *P* < 0.05). Two days later, there was no difference in Hp concentration between treatments (Fig. 1, *P* > 0.05). After weaning, ISCo pigs had the highest Hp concentration on d 2 (*P* < 0.01), while on d 4, Hp levels were higher in CW pigs than IS pigs and ISCo pigs were intermediate (Fig. 1, *P* = 0.01).

The concentration of glycerol in plasma was highest in CW piglets on d −6 and −4 before weaning (Table 9, *P* < 0.05 and < 0.01 respectively for d −6 and −4). Two days after weaning, there was no difference in glycerol concentration between treatments (Table 9, P > 0.05) and on the final blood sampling day (d 4), there was a tendency for ISCo pigs to have a lower glycerol concentration than CW pigs (Table 9, *P* < 0.1).

**Table 9:** Plasma glycerol concentrations in three different weaning regimens before and after weaning

Day^1,2^	Treatment^3^			*P*-value
	CW	IS	ISCo	
D −6	81^a^(68.0-95.7))	57^b^(46.1-69.3)	53^b^(42.8-65.2)	0.004
D −4	78^a^(61.7-95.3)	44^b^(32.4-57.8)	45^b^(33.2-59.0)	0.002
D 2	20 (12.4-29.5)	27 (16.4-40.1)	12 (6.7-19.9)	0.613
D 4	20^x^(13.9-28.1)	12^xy^(6.7-19.9)	10^y^(5.7-15.7)	0.053

^1^Day in relation to weaning with 0 representing weaning (e.g. -6 is 6 d before weaning). ^2^Data has been transformed via square root and then back transformed and expressed as least square means with 95% confidence intervals

^3^CW = conventional weaning (*n* = 10 pre-weaning, *n* = 9 post-weaning), IS = intermittent suckling (*n* = 10 pre-weaning, *n* = 9 post-weaning), ISCo = intermittent suckling with co-mingling (*n* = 10 pre-weaning, *n* = 9 post-weaning)

^ab^Values within a row not having the same superscript are significantly different

^xy^Values within a row with different superscripts are trends

## Discussion

Intermittent suckling systems mimic (semi) natural housing conditions where piglets do not have continuous access to the sow through lactation. From a production perspective, the potential benefits of IS are twofold: (i) periods of separation force piglets to explore nutrient options other than milk, stimulating an interest in creep feed [6, 7], and (ii) with enforced separation, sows may show oestrus meaning they can be mated while still lactating, which allows piglets to be weaned later [10] and gives them even more time to become familiar with solid food [8, 23]. The focus of the present study was to use two different IS regimes to stimulate feed intake and reduce post-weaning stress in piglets in order to improve post-weaning performance without an extended lactation. Co-mingling was used in addition to IS as a technique to reduce post-weaning aggression.

### Performance before and after weaning

In contrast to previous IS studies, IS for 8 h/d, 7 d before weaning in the current study did not increase creep feed intake during lactation compared with piglets continuously kept with the sow [6, 7, 23, 24]. Despite this result however, ADG and ADFI at 2 to 8 d after weaning were higher in IS pigs than in their CW counterparts. A similar outcome was reported by Berkeveld et al. [8] where piglets subjected to IS for 10 h/d, 7 d before weaning (26 d of age) had a comparable creep feed intake to CW piglets before weaning, but grew faster and ate more between 2 and 7 d after weaning. Also, Kuller et al. [6] reported that IS litters (12 h/d, 11 d before weaning) that had little or no creep feed intake during lactation still tended to have a higher weight gain after weaning than CW litters with a similar creep feed intake during lactation. Both authors hypothesised that IS litters were experiencing weaning as a less stressful event because there were already habituated with separation from the sow. The current study attempted to study this theory further by measuring behaviour and injury scores as well as plasma cortisol and Hp, two blood markers that are often increased at the time of weaning [25–29] (to be discussed in more detail below).

Socialising piglets in larger groups before weaning likely implements the social learning of eating behaviours with inexperienced eaters learning from experienced eaters [30]. In the current study, ISCo pigs ate the most creep feed during lactation, which was supported by the observations of ingestive-related behaviour on d −7. However, feed intake before weaning from d −17 to d −7 for both IS treatment groups tended to be higher than that of the controls. Therefore, in the case of ISCo, creep feed was already stimulated before the start of treatment intervention. This, in combination with the fact that a control litter in combination with CoM could not be included in the study, means that some caution must be used when interpreting the positive effects of the ISCo treatment.

Feed intake during lactation stimulates feed intake after weaning [31], and the higher creep feed intake for ISCo piglets during lactation was the likely cause of better growth and better feed intake in ISCoNF pigs 2 to 8 d after weaning compared with CW pigs. The fact that ISCoF pigs grew faster and tended to eat more solid feed than ISCoNF highlights the impact familiarity can have on piglet performance. Differences in how the ISCo piglets were grouped after weaning (ISCoF versus ISCoNF) only influenced the performance data and had no effects on behavioural data, injury scores or blood parameters measured in this study, which could have been due to the small sample size. Therefore, the mechanism by which familiarity improves post-weaning performance is not immediately obvious from the results of the current study. Nevertheless, a study by Kanitz et al. [32], where piglets were subjected to 4 h of maternal and littermate deprivation at different ages reported that the presence of an age-matched conspecific has a direct calming effect as measured by hypothalamic-pituitary- adrenal axis activity and behavioural responses in test situations. This was further influenced by the degree of familiarity between the piglets, with a familiar conspecific providing the most social support, allowing pigs to cope with stress better. Alternatively, other studies have shown that aggression between familiar dyads is lower than that between unfamiliar dyads [17, 33], with the exception of aggression around the feeder [34].

### Behavioural effects of IS and ISCoM before and after weaning

In the current study, ISCo piglets displayed the greatest proportion of total observations on aggressive behaviour on the first day of separation and mixing (d −7). This corresponds to results of other investigations [35–37]. In the current study, however and similar to results from Kanaan et al. [36], aggressive behaviour appeared to be transient with no difference between treatments for aggression or scratch scores closer to the time of weaning. The difference in available floor space for IS and ISCo piglets during times of separation from the sow (0.42 m^2^ for IS versus 0.21 m^2^ for ISCo) may have also been a confounding factor contributing to increased aggression [38]. After weaning, aggression was rarely observed across all treatments, which may have been partly due to the use of instantaneous behaviour sampling since events like the delivery of aggression are generally short in duration and continuous observations may be a more reliable sampling technique to capture the behaviour [18]. Injury scores including scratches and redness were also included in the current study as another way to measure aggression. Before weaning, injury scores were weakly correlated with aggressive behaviour, but after weaning, there was no correlation. This is most likely due to the low number of observations for aggression given other studies have shown skin lesions scores to be a reliable method to assess aggression [18, 39]. A reduced scratch and redness score in ISCo pigs 2 d after weaning combined with no difference in injury scores between ISCoF and ISCoNF may suggest that pre-weaning socialisation can teach piglets how to avoid fights with foreign pigs, be more tolerant of unfamiliar pigs, and (or) allow them to establish dominance hierarchies quicker through learnt social skills [16, 17, 36]. The reason CW piglets had a higher redness score than both IS treatments just before the event of weaning is not known. However, since there was no correlation between scratch and redness scores before weaning, the possibility of CW piglets rubbing up against each other in competition for the teat may have caused more redness at the time the injury scores were recorded.

Piglets subjected to IS with or without CoM during lactation showed a more than two-fold increase in percentage of scan samples in exploratory and play behaviour on the first day of separation compared with piglets that remained with their sow continuously. Similarly, Berkeveld et al. [40] reported a greater total activity at the start of IS when piglets were separated from the sow for 12 h per day. It was hypothesised that the increase in activity may have been due to restlessness associated with sudden and previous unexperienced separation from the sow because, similar to the present study, the behaviour reduced over time (d −7 to −1) and sleeping increased as IS and ISCo piglets became familiar with the process.

Manipulative behaviours such as belly nosing and manipulating pen or littermates can be signs of piglet distress [9]. To the authors’ knowledge, few studies have reported on manipulative behaviour in relation to CoM techniques. Alternatively, in an IS study by Berkeveld et al. [40], manipulative behaviour was considerably less in IS piglets compared with CW pigs on d 37 of the experiment, but this comparison was made at a time when CW pigs had been fully weaned, but the IS litters were still intermittently in contact with the sow. In the current study, IS piglets expressed the lowest level of manipulative behaviour compared with the other treatment groups before and after weaning suggesting that IS is not associated with the development of behaviour patterns indicative of piglet distress. In contrast, manipulative behaviour did not appear to dominate in the CW treatment more than the ISCo treatment before or after weaning, making it difficult to draw firm conclusions. On the day of weaning, while ISCo and CW piglets engaged in active behaviours such as manipulation, exploration/play, and ingestive-related behaviours (ISCo only), IS pigs spent virtually all their time sleeping in their pen. Other inactive behaviours including lying and sitting, which can be considered as symptoms of stress [41], were measured separately to sleeping in the current study. Therefore, the high level of sleeping observed in IS piglets on the day of weaning (2 h after the event) likely indicates that IS pigs were comfortable with their new surroundings [40, 42]. This, in combination with the reduced level of manipulative behaviour on the day of weaning, may have contributed to the improvement in post-weaning performance observed 2 to 8 d after weaning. In contrast, CW pigs had the highest level of sitting and lying behaviour on the day of weaning and d 7 after weaning, which could suggest that these pigs were experiencing more distress [9].

### Effects of IS and ISCo on bloods parameters before and after weaning

The possibility that CW pigs experienced more distress after weaning as suggested by the behavioural data, is not supported by the plasma cortisol results with no difference between treatments detected 2 d after weaning. This lack of difference in cortisol between treatments 2 d after weaning might be related to the timing of blood sampling. At weaning, cortisol levels have been shown to reduce over a 24 h period [43]. Therefore, taking a blood sample 2 d after the initial mixing period might have missed the maximum value. Pluske and Williams [20] and Turpin et al. [24] had similar outcomes sampling at two and one day after weaning, respectively. The reason why IS pigs had a higher cortisol concentration 4 d after weaning is unknown. However, the mean concentration of cortisol at d 4 after weaning for IS pigs (32 ng/mL) was not as high as levels associated with weaning associated stress in other studies [27, 44] (62 ng/mL and 41 ng/mL respectively). In contrast to the cortisol results, IS pigs had the lowest Hp concentrations on both measurement days after weaning. Haptoglobin is a major acute phase protein in the pig that serves as an integral part of the innate immune response, making it a suitable non-specific marker to monitor animal health and well-being [45]. While previous results from the research group have not reported an influence of IS on Hp values [24], post-weaning Hp results in the current study suggest that IS (without CoM) had an influence on the general wellness of pigs [46]. In saying this however, Hp values for all treatments remained below the acute range of 3,000 μg/mL to 8,000 μg/mL [47] throughout the experiment, suggesting that acute infection or inflammation was not likely present. Furthermore, the increase in Hp in IS pigs on d −6 may reflect the transient increase in stress associated with the beginning of IS as previously reported by Turpin et al. [24]. The reason why there was no increase in plasma Hp for ISCo piglets at the same time might have been related to the increase in creep feed intake for these piglets around this time.

Plasma glycerol was measured in this experiment to give an indication of lipid mobilisation under the three different weaning regimes. The CW piglets had the highest pre-weaning values, most likely reflecting a greater overall lipid metabolism as a result of lipid intake from milk. However, at 4 d after weaning, the tendency for glycerol levels to be higher in CW piglets compared with ISCo piglets most likely reflects a greater rate of lipolysis consistent with the differences in feed intake.

## Conclusion

These results show that an IS regime (without CoM) involving an 8 h/d separation for 7 d before weaning improved ADG and ADFI in the immediate post-weaning period compared with conventional weaning. However, this improvement did not seem to occur through increased familiarisation with creep feed, but rather through the prevention or attenuation of the weaning-associated stress response as evidenced by increased sleeping behaviour and reduced manipulative behaviour immediately after weaning as well as reduced post-weaning Hp levels. The addition of CoM to the IS regime also improved post-weaning performance between 2 and 8 d after weaning most likely due to social learning facilitating more eating before weaning and reduced aggression after weaning also reducing stress, as evidenced by a reduction post-weaning injury scores. Furthermore, grouping familiar pigs together after weaning additively improved ADG, ADFI and FCR between 2 and 8 d after weaning. Overall, these results suggest that mimicking certain aspects of weaning under natural conditions, such as gradual maternal separation and the opportunity to mix with non-litter mates in lactation, can positively affect post-weaning performance, and highlights opportunities for potential housing systems that enhance piglet welfare whilst also potentially facilitating mating in lactation.

## Abbreviations

ADFI: Average daily feed intake
ADG: Average daily gain
BW: Body weight
CoM: Co-mingling
FCR: Feed conversion ratio
GIT: Gastrointestinal tract
Hp: Haptoglobin
IM: Intramuscular
IS: Intermittent suckling.

## References

[1] Pajor EA, Fraser D, Kramer DL (1991). Consumption of solid food by suckling pigs: individual variation and relation to weight gain. Appl Anim Behav Sci.

[2] Leibbrandt VD, Ewan RC, Speer VC, Zimmerman DR (1975). Effect of weaning and age at weaning on baby pig performance. J Anim Sci.

[3] Brooks P, Tsourgiannis C, Pluske JR, Le Dividich J, Verstegen MWA (2003). Factors affecting the voluntary feed intake of the weaned pig. Weaning the pig: concepts and consequences.

[4] Pluske JR, Hampson DJ, Williams IH (1997). Factors influencing the structure and function of the small intestine in the weaned pig: a review. Livest Prod Sci.

[5] Pié S, Lalles J, Blazy F, Laffitte J, Sève B, Oswald I (2004). Weaning is associated with an upregulation of expression of inflammatory cytokines in the intestine of piglets. J Nutr.

[6] Kuller W, Soede N, van Beers-Schreurs H, Langendijk P, Taverne M, Verheijden J (2004). Intermittent suckling: effects on piglet and sow performance before and after weaning. J Anim Sci.

[7] Kuller W, Soede N, van Beers-Schreurs H, Langendijk P, Taverne M, Kemp B (2007). Effects of intermittent suckling and creep feed intake on pig performance from birth to slaughter. J Anim Sci.

[8] Berkeveld M, Langendijk P, Soede NM, Kemp B, Taverne MA, Verheijden JH (2009). Improving adaptation to weaning: effect of intermittent suckling regimens on piglet feed intake, growth, and gut characteristics. J Anim Sci.

[9] Dybkjær L (1992). The identification of behavioural indicators of ‘stress’ in early weaned piglets. Appl Anim Behav Sci.

[10] Kemp B, Soede N (2012). Should weaning be the start of the reproductive cycle in hyper‐prolific sows? a physiological view. Reprod Domest Anim.

[11] Weary DM, Pajor EA, Bonenfant M, Fraser D, Kramer DL (2002). Alternative housing for sows and litters: part 4. Effects of sow-controlled housing combined with a communal piglet area on pre-and post-weaning behaviour and performance. Appl Anim Behav Sci.

[12] van Nieuwamerongen S, Soede N, van der Peet-Schwering C, Kemp B, Bolhuis J (2015). Development of piglets raised in a new multi-litter housing system vs. Conventional single-litter housing until 9 weeks of age. J Anim Sci.

[13] Pajor EA, Weary DM, Fraser D, Kramer DL (1999). Alternative housing for sows and litters: part 1. Effects of sow-controlled housing on responses to weaning. Appl Anim Behav Sci.

[14] Petersen H, Vestergaard K, Jensen P (1989). Integration of piglets into social groups of free-ranging domestic pigs. Appl Anim Behav Sci.

[15] Kutzer T, Bünger B, Kjaer JB, Schrader L (2009). Effects of early contact between non-littermate piglets and of the complexity of farrowing conditions on social behaviour and weight gain. Appl Anim Behav Sci.

[16] D’Eath RB (2005). Socialising piglets before weaning improves social hierarchy formation when pigs are mixed post-weaning. Appl Anim Behav Sci.

[17] Li Y, Wang L (2011). Effects of previous housing system on agonistic behaviors of growing pigs at mixing. Appl Anim Behav Sci.

[18] Verdon M, Morrison RS, Hemsworth PH (2016). Rearing piglets in multi-litter group lactation systems: effects on piglet aggression and injuries post-weaning. Appl Anim Behav Sci.

[19] Widowski T, Cottrell T, Dewey C, Friendship R (2003). Observations of piglet-directed behavior patterns and skin lesions in eleven commercial swine herds. J Swine Health Prod.

[20] Pluske J, Williams I (1996). Reducing stress in piglets as a means of increasing production after weaning: administration of amperozide or co-mingling of piglets during lactation?. J Anim Sci.

[21] Bolhuis JE, Schouten WGP, Schrama JW, Wiegant VM (2005). Behavioural development of pigs with different coping characteristics in barren and substrate-enriched housing conditions. Appl Anim Behav Sci.

[22] Eckersall P, Duthie S, Safi S, Moffatt D, Horadagoda N, Doyle S (1999). An automated biochemical assay for haptoglobin: prevention of interference from albumin. Comp Haematol Int.

[23] Berkeveld M, Langendijk P, van Beers-Schreurs HM, Koets AP, Taverne MA, Verheijden JH (2007). Postweaning growth check in pigs is markedly reduced by intermittent suckling and extended lactation. J Anim Sci.

[24] Turpin DL, Langendijk P, Chen T-Y, Lines D, Pluske JR (2016). Intermittent suckling causes a transient increase in cortisol that does not appear to compromise selected measures of piglet welfare and stress. Animals.

[25] van der Meulen J, Koopmans S, Dekker R, Hoogendoorn A (2010). Increasing weaning age of piglets from 4 to 7 weeks reduces stress, increases post-weaning feed intake but does not improve intestinal functionality. Animal.

[26] Worsaae H, Schmidt M (1980). Plasma cortisol and behaviour in early weaned piglets. Acta Vet Scand.

[27] Moeser AJ, Vander Klok C, Ryan KA, Wooten JG, Little D, Cook VL (2007). Stress signaling pathways activated by weaning mediate intestinal dysfunction in the pig. Am. J. Of physiol. Gastrointest. Liver Physiol.

[28] Sauerwein H, Schmitz S, Hiss S (2005). The acute phase protein haptoglobin and its relation to oxidative status in piglets undergoing weaning-induced stress. Redox Rep.

[29] Capozzalo MM, Kim JC, Htoo JK, de Lange CF, Mullan BP, Hansen CF (2015). Effect of increasing the dietary tryptophan to lysine ratio on plasma levels of tryptophan, kynurenine and urea and on production traits in weaner pigs experimentally infected with an enterotoxigenic strain of escherichia coli. Arch Anim Nutr.

[30] Morgan C, Lawrence A, Chirnside J, Deans L (2001). Can information about solid food be transmitted from one piglet to another?. J Anim Sci.

[31] Bruininx E, Binnendijk G, Van der Peet-Schwering C, Schrama J, Den Hartog L, Everts H (2002). Effect of creep feed consumption on individual feed intake characteristics and performance of group-housed weanling pigs. J Anim Sci.

[32] Kanitz E, Hameister T, Tuchscherer M, Tuchscherer A, Puppe B (2014). Social support attenuates the adverse consequences of social deprivation stress in domestic piglets. Horm Behav.

[33] Stookey JM, Gonyou HW (1998). Recognition in swine: recognition through familiarity or genetic relatedness?. Appl Anim Behav Sci.

[34] Puppe B (1998). Effects of familiarity and relatedness on agonistic pair relationships in newly mixed domestic pigs. Appl Anim Behav Sci.

[35] Parratt CA, Chapman KJ, Turner C, Jones PH, Mendl MT, Miller BG (2006). The fighting behaviour of piglets mixed before and after weaning in the presence or absence of a sow. Appl Anim Behav Sci.

[36] Kanaan VT, Pajor EA, Lay DC, Richert BT, Garner JP (2008). A note on the effects of co-mingling piglet litters on pre-weaning growth, injuries and responses to behavioural tests. Appl Anim Behav Sci.

[37] Wattanakul W, Stewart A, Edwards S, English P (1997). Effects of grouping piglets and changing sow location on suckling behaviour and performance. Appl Anim Behav Sci.

[38] Hvozdik A, Kottferova J, Da Silva Alberto J (2002). Ethological study of social behaviour of pigs from the point of view of housing restriction. Archiv Fur Tierzucht.

[39] Turner SP, Farnworth MJ, White IM, Brotherstone S, Mendl M, Knap P (2006). The accumulation of skin lesions and their use as a predictor of individual aggressiveness in pigs. Appl Anim Behav Sci.

[40] Berkeveld M, Langendijk P, Bolhuis JE, Koets AP, Verheijden JH, Taverne MA (2007). Intermittent suckling during an extended lactation period: effects on piglet behavior. J Anim Sci.

[41] Colson V, Orgeur P, Foury A, Mormède P (2006). Consequences of weaning piglets at 21 and 28 days on growth, behaviour and hormonal responses. Appl Anim Behav Sci.

[42] Morgan T, Pluske J, Miller D, Collins T, Barnes AL, Wemelsfelder F (2014). Socialising piglets in lactation positively affects their post-weaning behaviour. Appl Anim Behav Sci.

[43] Rault J-L, Dunshea FR, Pluske JR (2015). Effects of oxytocin administration on the response of piglets to weaning. Animals.

[44] Moeser AJ, Ryan KA, Nighot PK, Blikslager AT (2007). Gastrointestinal dysfunction induced by early weaning is attenuated by delayed weaning and mast cell blockade in pigs. Am J Physiol Gastrointest Liver Physiol.

[45] Cray C, Zaias J, Altman NH (2009). Acute phase response in animals: a review. Comp Med.

[46] Knura S, Lipperheide C, Petersen B, Wendt M, Tielen MJM, Voets MT (2000). Impact of hygienic environment on haptoglobin concentration in pigs. Proceedings of the 10th international congress on animal hygiene.

[47] Sales N, Collins D, Collins A, McKenna T, Bauer M, Parke C (2015). Porcine haptoglobin levels measured at 7–14 days after weaning were independent of age, weight or gender. Anim Prod Sci.

